# Colo-Rectitis

**Published:** 1877-09

**Authors:** J. G. Westmoreland

**Affiliations:** Atlanta, Ga.


### COLO-RECTITIS.

By J. G. WESTMORELAND, M.D.

Bowel affections take special names according to the part diseased—names which indicate, not only the particular location, but the pathological condition, also. Other names, indicating some prominent symptoms, are often used—I may say almost exclusively—for the various forms of bowel disturbance. Diarrhœa, for example, simply indicating frequent, copious flow from the bowels, is used for the name of the disease or disturbance, whether it be irritation, acute or chronic, inflammation of the mucous surface of the bowels, or unusual effusion into the canal from other cause. The name only has reference to the free discharges from any of the causes mentioned, and has, therefore, no pathological nor therapeutical significance. Diarrhœa from acute inflammation of the alimentary mucous membrane, is very different, therapeutically, from diarrhœa caused by copious watery exhalations into the canal.

The same error is prevalent in regard to the disease which heads this article. Dysentery, a term indicating painful, difficult discharges, from any cause, is the name commonly applied to inflammation of the rectum and lower portion of the colon. This dysentery — difficulty in evacuations — is a prominent symptom of inflammation in these parts, and, although it may be the result of other forms of disturbance, the term is very generally used when the inflammatory condition exists. Mere irritation of the rectum, causing unusual spasmodic contractions, which produce this symptom, may with equal propriety be called dysentery. With those who conclude that this symptom is sufficient indication of the disease, and that the same remedy or plan of treatment is always proper in difficult evacuations, this error is particularly mischievous. A grain of opium which has effectually controlled the dysentery arising from irritation of the lower part of the canal, will be found insufficient to cure acute inflammation of the organ.

Moreover, this symptom may be constantly present, when the disease extends much beyond the parts mentioned. Even gastro-enteritis may be accompanied with distressing tenesmus, on account of inflammation in the rectum, also. It must be remembered, too, that there are various degrees of inflammation, as regards its violence and extension into the sub-mucous tissues; all of which produce violent dysentery, but demand more or less vigorous treatment, according to its activity and extension.

During summer and autumn, mucous inflammation of the rectum and colon sometimes prevails extensively, assuming the magnitude of an epidemic. Such form of the disease will be found less under control of ordinary means. Opium will, however, generally be found a suitable test of intensity, under such circumstances. In dysentery from temporary irritation, or slight inflammation, limited in extent, one-fourth grain of morphine will be found sufficient to allay all the dysenteric symptoms for several hours, while in violent inflammation, such as attends the epidemic form, the frequent paroxysms are not arrested by opiates. In both, however, the suffering and frequent evacuations are equally distressing to the patient. Not only so, but bloody and mucous discharges, in slight and superficial inflammation of the rectum, are about the same as when the disease is more extensive and violent.

A knowledge of the cause of colo-rectitis certainly should give decided therapeutic advantages. If torpor of the liver, leading to imperfect circulation through the portal vessels, should be the prime factor in its production, as some suppose, the treatment ought, of course, to include means calculated to relieve this difficulty. Whether the more violent cases of the disease depend upon this cause or not, it seems quite reasonable to conclude that sporadic cases, at any rate are often relieved by means tending to restore activity to the liver. Moreover, the fact that violent epidemic colo-rectitis is not usually controlled by such means alone, is not positive evidence against this opinion, because it is a well known fact that subsidence of the cause does not always lead to relief of the inflammation. The cause should not, of course, be suffered to continue, but when the effect has been decided, other means are necessary. Traumatic inflammation often attains such a degree of activity that removal of the ball or other exciting cause is not sufficient for its arrest.

In consideration of these facts it is not at all surprising that very different means are necessary in the treatment of various forms and degrees of disease producing the symptom dysentery.

As above stated, very simple means sometimes promptly relieve very distressing tenesmus tormina and bloody discharges, owing to the inflammation being slight and limited in extent. A pill, composed of tannin, opium and blue mass, repeated as circumstances require, leaves no further trouble in many cases. When, however, this course does not afford even temporary, decided relief, a continuance of the mercury beyond a few days does not seem to be called for. Indeed when from the violence and persistent character of the inflammation great prostration is likely to insue, mercurials continued are no doubt injurious, and should be substituted by nutritious and supporting means. While astringents, opiates, demulcents and counter-irritants are continued to lessen the local inflammation, the final prostration likely to result must be anticipated by the use of nourishing soups, milk and other kinds of unirritating fluid diet, and the support of nervous power by alcohol in addition to opium. Blistering should not be deferred beyond the time that the persistent character of the disease is determined, by the failure of opium to arrest, for several hours, the discharges.

Saline cathartics are doubtless serviceable at the commencement of the disease, by the depleting effect thus had on the engorged blood-vessels of the bowels, and the removal of hardened irritating fæces which sometimes remain above the diseased portion. The too long continuance of their use, however, must necessarily add to, instead of lessening the existing inflammation, by keeping up constant agitation of the inflamed part.

We cannot speak too highly of the local application of opiates, astringents and demulcents, by injection into the rectum, while they are being used by the mouth. When given in this way they come in contact with the inflamed mucous membrane, while in full strength; and we have witnessed very prompt relief in this way from the unpleasant symptoms when they had resisted their use by the stomach.

